# Molecular diversity in isocitrate dehydrogenase-wild-type glioblastoma

**DOI:** 10.1093/braincomms/fcae108

**Published:** 2024-03-27

**Authors:** Jawad Fares, Yizhou Wan, Richard Mair, Stephen J Price

**Affiliations:** Academic Neurosurgery Division, Department of Clinical Neurosciences, University of Cambridge, Cambridge, CB2 0QQ, UK; Cambridge Brain Tumour Imaging Laboratory, Division of Neurosurgery, Department of Clinical Neurosciences, University of Cambridge, Cambridge, CB2 0QQ, UK; Department of Neurological Surgery, Feinberg School of Medicine, Northwestern University, Chicago, IL 60611, USA; Academic Neurosurgery Division, Department of Clinical Neurosciences, University of Cambridge, Cambridge, CB2 0QQ, UK; Cambridge Brain Tumour Imaging Laboratory, Division of Neurosurgery, Department of Clinical Neurosciences, University of Cambridge, Cambridge, CB2 0QQ, UK; Academic Neurosurgery Division, Department of Clinical Neurosciences, University of Cambridge, Cambridge, CB2 0QQ, UK; Academic Neurosurgery Division, Department of Clinical Neurosciences, University of Cambridge, Cambridge, CB2 0QQ, UK; Cambridge Brain Tumour Imaging Laboratory, Division of Neurosurgery, Department of Clinical Neurosciences, University of Cambridge, Cambridge, CB2 0QQ, UK

**Keywords:** glioblastoma, IDH-wild-type, heterogeneity, neuroimaging, machine learning

## Abstract

In the dynamic landscape of glioblastoma, the 2021 World Health Organization Classification of Central Nervous System tumours endeavoured to establish biological homogeneity, yet isocitrate dehydrogenase-wild-type (IDH-wt) glioblastoma persists as a tapestry of clinical and molecular diversity. Intertumoural heterogeneity in IDH-wt glioblastoma presents a formidable challenge in treatment strategies. Recent strides in genetics and molecular biology have enhanced diagnostic precision, revealing distinct subtypes and invasive patterns that influence survival in patients with IDH-wt glioblastoma. Genetic and molecular biomarkers, such as the overexpression of neurofibromin 1, phosphatase and tensin homolog and/or cyclin-dependent kinase inhibitor 2A, along with specific immune cell abundance and neurotransmitters, correlate with favourable outcomes. Conversely, increased expression of epidermal growth factor receptor tyrosine kinase, platelet-derived growth factor receptor alpha and/or vascular endothelial growth factor receptor, coupled with the prevalence of glioma stem cells, tumour-associated myeloid cells, regulatory T cells and exhausted effector cells, signifies an unfavourable prognosis. The methylation status of O^6^-methylguanine–DNA methyltransferase and the influence of microenvironmental factors and neurotransmitters further shape treatment responses. Understanding intertumoural heterogeneity is complemented by insights into intratumoural dynamics and cellular interactions within the tumour microenvironment. Glioma stem cells and immune cell composition significantly impact progression and outcomes, emphasizing the need for personalized therapies targeting pro-tumoural signalling pathways and resistance mechanisms. A successful glioblastoma management demands biomarker identification, combination therapies and a nuanced approach considering intratumoural variability. These advancements herald a transformative era in glioblastoma comprehension and treatment.

## Teaser

Fares *et al*. explore tumoural heterogeneity in IDH-wild-type glioblastoma, unveiling genetic, epigenetic and microenvironmental factors shaping tumour dynamics and clinical outcomes.

## Graphical Abstract

**Graphical abstract:**
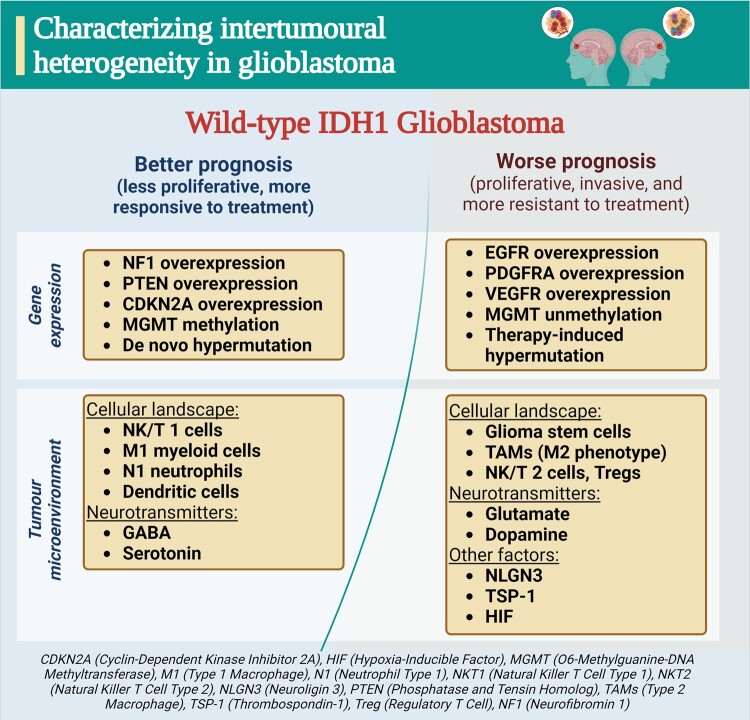

## Introduction

Glioblastoma remains an unmet challenge. Survival rates continue to be dismal with median overall survival ranging between 14 and 21 months despite neurosurgical resection, chemotherapy and radiotherapy in newly diagnosed glioblastoma.^1,2^ The heterogeneity of glioblastoma yields cellular resistance to therapy. As such, tumours recur and portend a median overall survival of 9–11 months only.^3,4^ Understanding the genetic and molecular nature of glioblastoma is essential for proper design of diagnostic and therapeutic strategies that can target this malignant disease.

Glioblastomas display considerable heterogeneity. Intertumoural heterogeneity refers to the differences between tumours from diverse patients.^5^ This intertumoural heterogeneity in glioblastoma is due to the epigenetic, genetic, protein and microenvironmental changes that make glioblastoma in one patient different from another. This inter-lesion diversity provides select tumours with unique functions that allow them to grow aggressively, survive hypoxic stresses and resist chemotherapy.^6^

The glioma classification system was initially based on distinguishing between expansive and infiltrative growth patterns, with infiltrative growth indicating malignancy.^7^ However, this classification did not fully capture glioma proliferation, including primary systematic diffuse or multicentric neoplastic growth. Subsequently, histopathology-based grading systems were developed to improve glioma classification by evaluating nuclear atypia, mitotic count, endothelial proliferation and necrosis.^8^ Over the past two decades, major advances helped establish different prognostic subtypes of glioblastoma, utilizing gene expression and transcriptomic analyses.^9,10^

The 2021 World Health Organization central nervous system tumour classification was a major step to improve the way brain tumours are diagnosed.^11^ Previous classifications focused on light microscopic changes and were limited by observational discrepancies reported by individual neuropathologists, leading to inconsistencies in responses to therapy and outcomes. This hindered clinical trial progress and the development of effective therapies. By focusing on the molecular characteristics, brain tumours could be better grouped into more homogeneous entities. This allows the development of targeted therapies that can help patients with glioblastoma.

**Figure 1:**
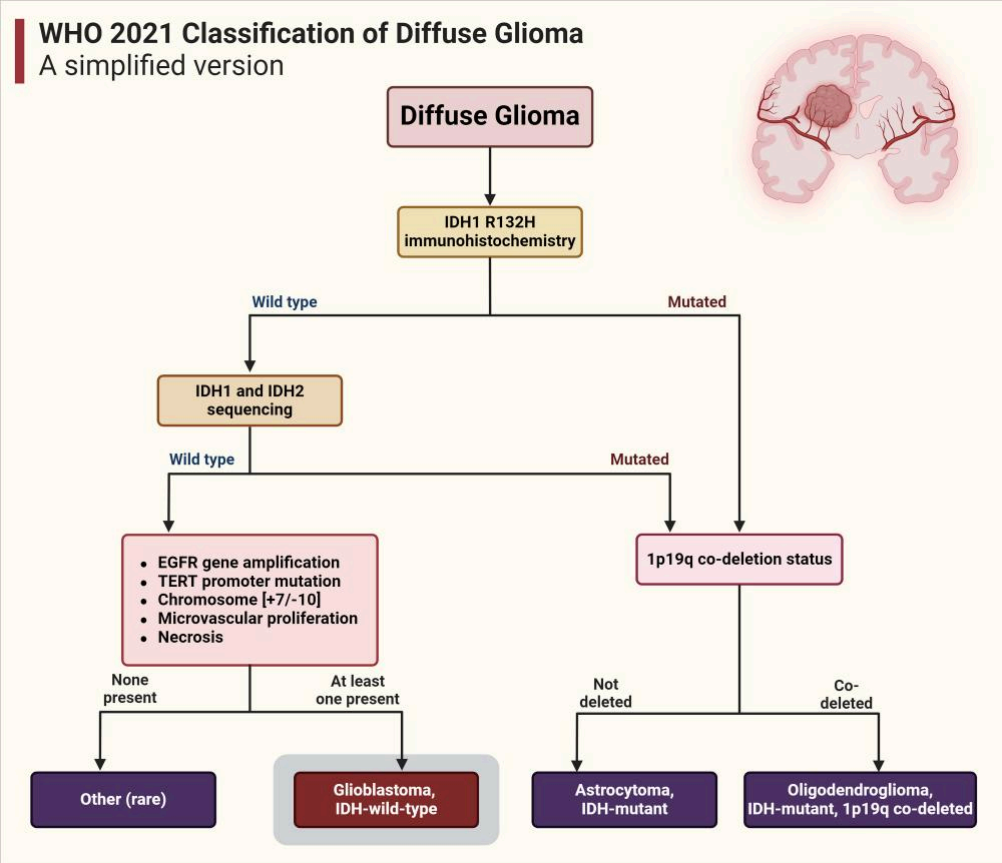
**Simplified version of the World Health Organization 2021 classification of diffuse glioma**. For the diagnosis of IDH-wild-type glioblastoma, tumour cell has to have an IDH wild-type phenotype, and at least one of the following: EGFR gene amplification, telomerase reverse transcriptase promoter mutation, chromosome +7/−10, angiogenesis and/or necrosis.

According to the 2021 World Health Organization classification, wild-type isocitrate dehydrogenase 1 (IDH1-wt) gliomas are categorized as glioblastomas, exhibiting distinct genomic alterations and high somatic mutation rates^11^ (Fig. 1). IDH enzymes are essential for the oxidative decarboxylation of isocitrate in the citric acid cycle, maintaining cellular homeostasis.^12^ These enzymes convert isocitrate to alpha-ketoglutarate using nicotinamide adenine dinucleotide phosphate (NADP+). In glioma cases, *IDH* genes can be mutated in 73% of patients, with the most common mutation occurring at arginine 132 (R132).^13^ These mutated IDH enzymes have distinct metabolic and epigenetic characteristics and respond differently to treatments,^14^ leading to improved patient prognoses compared to IDH1-wt. Nevertheless, only IDH-wt gliomas, regardless of their histological grade (i.e. the presence of necrosis or microvascular proliferation) can be classified as molecular glioblastoma.^15^ The new World Health Organization classification rendered IDH-mutant Grade 4 histological gliomas as separate Grade 4 astrocytoma.^15^

**Figure 2:**
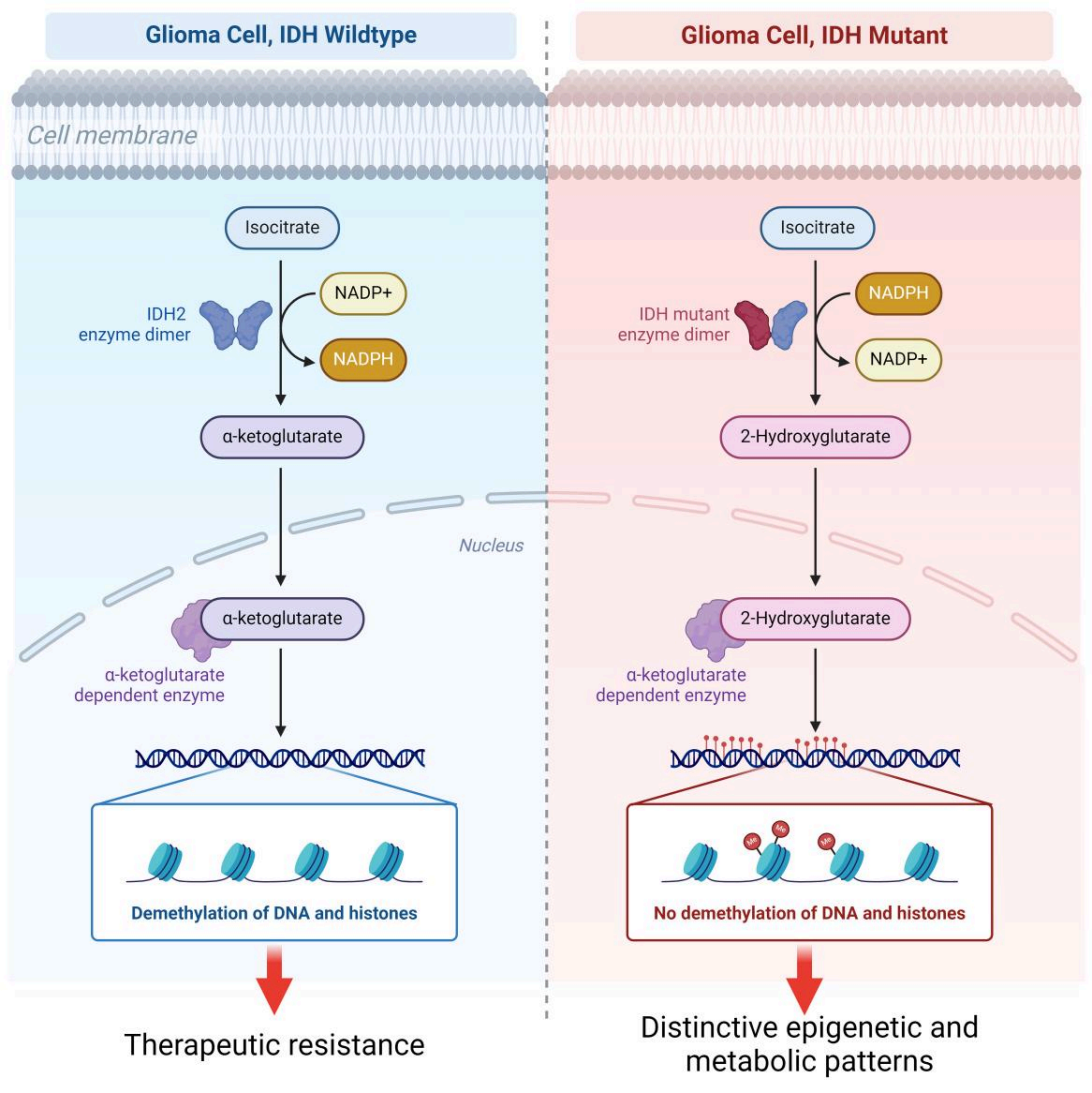
**Expression of IDH in glioblastoma**. IDH enzymes maintain cellular homeostasis by converting isocitrate to alpha-ketoglutarate. IDH mutations cause alpha-ketoglutarate to be converted to 2-hydroxyglutarate. Accumulation of 2-hydroxyglutarate causes the methylation of DNA and histones, leading to distinct metabolic and epigenetic characteristics, as well as a differential response to therapeutic interventions. IDH-mutated gliomas possess unique genetic and clinical features, with patients displaying improved prognoses compared to those with wild-type IDH genes. *Nicotinamide adenine dinucleotide phosphate (NADPH)*.

IDH1-wt glioblastoma typically displays distinct molecular features that contribute to its aggressive nature (Fig. 2). Genetic alterations, such as telomerase reverse transcriptase promoter mutation, epidermal growth factor receptor (*EGFR*) amplification and combined chromosome 7 gain/chromosome 10 loss (+7/−10),^16-18^ are associated with poor clinical outcomes, even in lower-grade (Grade 2 or 3) histopathology.^17,19-23^ Mutations in tumour suppressor genes such as tumour protein P53 (*TP53*), phosphatase and tensin homolog (*PTEN*) and cyclin-dependent kinase inhibitor 2A/B (*CDKN2A/B*) are commonly observed, along with dysregulation of pathways involved in oncogenesis, such as EGFR amplification and aberrant RTK–RAS–PI3K signalling.^9,15,24^ In addition, IDH1-wt glioblastomas demonstrate ATP-dependent helicase ATRX (ATRX) activity, which is associated with alternative lengthening of telomeres and epigenetic regulation.^25^ IDH1-wt glioblastomas are classified as glioma CpG island methylator phenotype low tumours and display alterations in retinoblastoma (*RB*), *TP53* and *PTEN*, deletions in *CDKN2A/B* and amplification of *CDK4*.^26-28^

This review explores the molecular and microenvironmental determinants of prognosis and therapeutic response in IDH-wt glioblastoma. Recognition of intertumoural heterogeneity within glioblastoma is imperative to design clinical trials and develop targeted therapies that can benefit patients in the clinical setting.

## Genetic and molecular signatures

Glioblastoma was among the first cancer types examined by the Cancer Genome Atlas (TCGA). By means of sequencing analysis, TCGA identified somatic alterations in *TP53* (78%), *RB1* (87%) and RTK/RAS/PI3K signalling pathways (88%), with these alterations present in 74% of tumours^29,30^ (Table 1). Three transcriptional subtypes, proneural, mesenchymal and proliferative, or four subtypes, proneural, neural, mesenchymal and classical, have been identified in high-grade gliomas, each characterized by distinct molecular alterations.^44,45^ The mesenchymal subtype was characterized by high chitinase-3-like protein 1/YKL40 expression and neurofibromin 1 (*NF1*) deletions, and it exhibits worse survival outcomes.^41-44^ The classical subtype was marked by chromosome 7 gain, chromosome 10 loss, *EGFR* amplification and EGFRvIII expression.^45,47^ The proneural subtype expresses OLIG2 and is associated with secondary glioblastoma, younger age and mutations in *PDGFRA*, *CDK4*, *TP53* and *IDH*.^44-46,47^ The neural subtype may be an artifact caused by non-tumour cells.^41^ Immunohistochemistry-based profiling^48-50^ and machine learning approaches^51-53^ have been employed to correlate molecular and protein-based classifications of glioblastoma, achieving concordance between transcriptomics and immunohistochemistry and enabling the detection of tumour infiltration regions. However, this classification remained prone to sampling bias, with evidence suggesting that different subtypes can exist in a single tumour. Using synthetic genetic tracing cassettes, proneural glioblastoma was found to be intrinsic, while mesenchymal glioblastoma was adaptable, influenced by inflammation, differentiation cues and DNA damage, driven by immune cells into a therapeutic-resistant transition.^54^

**Table 1:** Intertumoural heterogeneity in glioblastoma through epigenetic and molecular signatures

Gene	Expression/status	Impact	Ref
IDH	Wild-type	Increased chromatin remodelling, lengthening of telomeres, inactivation of tumour suppressors and increased pro-tumourigenic macrophages in the tumour microenvironment	^9, 15, 24, 25^
TERT	Mutation	Alters telomerase reverse transcriptase gene promoter and promotes telomerase reactivation	^31^
PTEN	Promoter hypermethylation/loss	Activates the PI3K pathway, leading to cancer cell proliferation, adhesion and invasion	^28, 32, 33^
CDKN2A/B	Promoter hypermethylation, deletion/loss	Promotes the proliferation of glioblastoma stem cells	^34^
RB	Promoter hypermethylation/loss	Promotes glioma cell proliferation and resistance to CDK4/6 inhibitors	^35-37^
EGFR	Amplification	Inhibits apoptosis and contributes to angiogenesis and aggressiveness	^38, 39^
PDGFR	Overexpression	Increases glioblastoma cellular proliferation	^40^
NF1	Deactivation	Increases glioma stemness and increases chemotaxis and infiltration of TAMs and microglia in the immune microenvironment	^41^
MGMT	Hypomethylation	Destabilize chromosomal integrity and promote resistance to temozolomide	^42, 43^

### Epidermal growth factor receptor expression status

**Figure 3:**
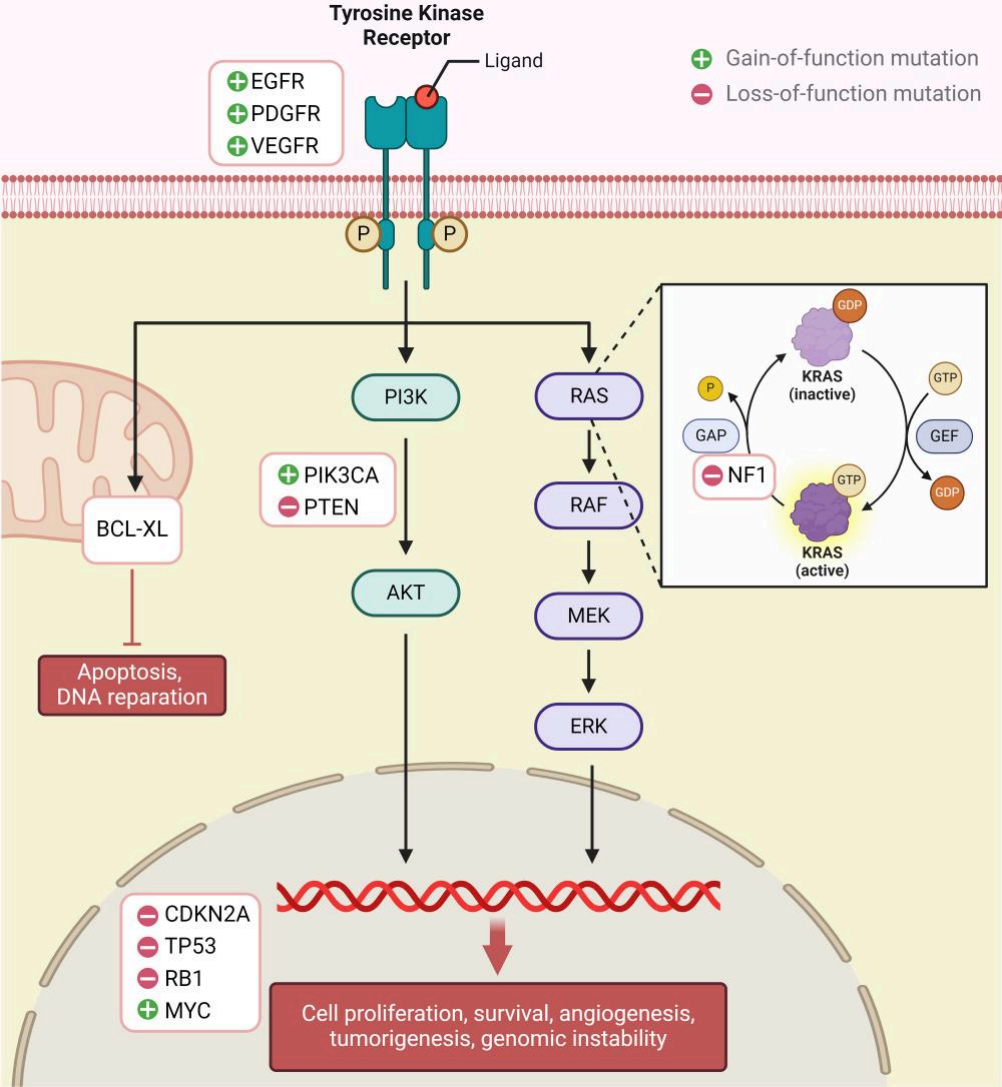
**Oncogenes versus tumour suppressor genes in glioblastoma**. EGFR is frequently mutated in glioblastoma and constitutively activates several signalling pathways, inhibits apoptotic cell death and increases growth and invasiveness of glioblastoma cells. PDGF and PDGFR are overexpressed in glioblastomas, particularly, PDGFRA. The overexpression of PDGFRA is associated with more aggressive phenotypes and poorer survival outcomes. The NF1 gene encodes neurofibromin, which inhibits the RAS/MAPK signalling pathway. Inactivation of two NF1 alleles is required for glioma formation. *Vascular endothelial growth factor receptor (VEGFR), KRAS (Kirsten rat sarcoma), RAS (rat sarcoma), RAF (rapidly accelerated fibrosarcoma), MEK (mitogen-activated protein kinase), ERK (extracellular signal-regulated kinase), PI3K (phosphoinositide 3-kinase), AKT (protein kinase B), PTEN (phosphatase and tensin homolog), BCL-XL (B-cell lymphoma-extra large), RB1 (retinoblastoma 1), MYC (myelocytomatosis oncogene), GAP (GTPase-activating protein), GDP (guanosine diphosphate), GTP (guanosine triphosphate) and GEF (guanine nucleotide exchange factor)*.

*EGFR* is among the genes that are frequently mutated in glioblastoma, where it is amplified in 50% of all glioblastomas.^24,55-57^ Specifically, the in-frame deletion of exons 2–7 (*EGFRvIII*) is its most common genomic mutation.^38^ EGFRvIII classically arises after chromosome 7 amplification and EGFR overexpression.^58^ While *EGFR* amplification is an early genetic aberration acquired early in glioblastoma tumorigenesis, EGFRvIII amplification occurs upon tumour progression and contribute to angiogenesis and aggressiveness.^39^ It constitutively activates the receptor independent of ligand binding,^59^ triggering PI3K/AKT and RAS/RAF/MEK/ERK signalling pathways and STAT3 activation.^60^ It plays a key role in inhibiting apoptotic cell death and increasing growth and invasiveness of glioblastoma cells^38^ (Fig. 3). Yet, the expression of EGFRvIII varies across time, where its expression decreases after initial resection,^56,61,62^ and can be lost as mechanism of resistance to therapy, reappearing again when therapy is stopped.^63^ EGFRvIII expressing cells can retain their EGFR-wild-type status via paracrine mechanisms and IL-6-driven signalling.^64^ This raises questions over the presumed role of EGFRvIII as a driver mutation for glioblastoma and makes it harder for targeted therapies to be effective. To date, anti-EGFRvIII therapies, such as rindopepimut, have failed to improve clinical outcomes.^65^ Further investigation showed that the glioma cells lost EGFRvIII antigen expression to escape immunogenic attacks induced by the administered vaccine,^66^ hinting that EGFRvIII expression exhibits mosaicism and that different pathways could be at play to drive glioma growth. Using computational methods, EGFR was found to be co-amplified with its active enhancer elements on circular extrachromosomal DNA. These enhancer elements impacted regulatory elements and chromatin structure within glioblastoma.^67^ Extrachromosomal DNA is present in almost half of human cancers due to uneven segregation of chromosomes without a centromere.^68^ Mutant EGFR extrachromosomal DNA is eliminated during anti-EGFR treatment, causing resistance, yet reappears after drug cessation.^63^

### Platelet derived growth factor receptor expression status

Growth factors preserve the proliferation and self-renewal of cells. Often, this process is disrupted in cancer. Platelet-derived growth factors (PDGF) and their receptors (PDGFR) are overexpressed in gliomas. *PDGFRA* expression inform intertumoural heterogeneity.^69^ It is a critical gene encoding the second most mutated tyrosine kinase receptor, after EGFR, in glioblastoma^24,45^ (Fig. 3). Overexpression of PDGFRA is associated with more malignant phenotypes and older age, leading to poorer survival outcomes in glioblastoma.^70,71^ A specific c.1403A>G mutation in exon 10 of *PDGFRA* can lead to glioblastoma cells that exhibit higher proliferation through the PDGFRA and CDK4–CDK6/cyclin D1 signalling pathways.^40^ As such, targeting these pathways with kinase inhibitors can be a promising therapeutic strategy in glioblastoma. PDGFRA can further activate the AKT pathway through hypoxia-inducible factor 1-alpha (HIF1A), leading to increased glioblastoma proliferation and contributing to its malignancy.^72^ Drugs, such as imatinib, sorafenib, nilotinib and sunitinib, have been developed to target PDGFRA in the tumour setting.^43^ Nevertheless, agents targeting PDGFRA have, so far, been found unsuitable due to toxicity and/or disease progression despite treatment.^73-77^

### Neurofibromin 1 expression status

NF1 gene expression encodes neurofibromin that is found in neurons and astrocytes within the central nervous system. Neurofibromin plays a key role in controlling intracellular growth pathways by inhibiting the RAS/MAPK signalling pathway and increasing the levels of cyclic adenosine monophosphate within the cell^78-80^ (Fig. 3). This occurs through the regulation of FOS-like 1 expression, an AP-1 transcription factor, that encodes for FOS-related antigen 1 and positively corelates with tumour progression and worse prognosis in glioblastoma.^81,82^ The inhibition of FOS-like 1 by NF1 inhibits cellular growth and stemness. As a tumour suppressor gene, the inactivation of both alleles of *NF1* are necessary for the loss of neurofibromin and subsequent tumour formation.^83,84^ NF1 deactivation is associated with the chemotaxis and infiltration of tumour-associated macrophages (TAMs) and microglia in the immune microenvironment of IDH-wt glioblastomas.^41^ Longitudinal transcriptomic analysis showed that this phenotype of expression is present in 55% of glioblastoma cases.^41^ The population size of M2 macrophages in the immune microenvironment was further associated with relapse post radiotherapy and tumour resistance.^41^

### DNA methylation status

**Figure 4:**
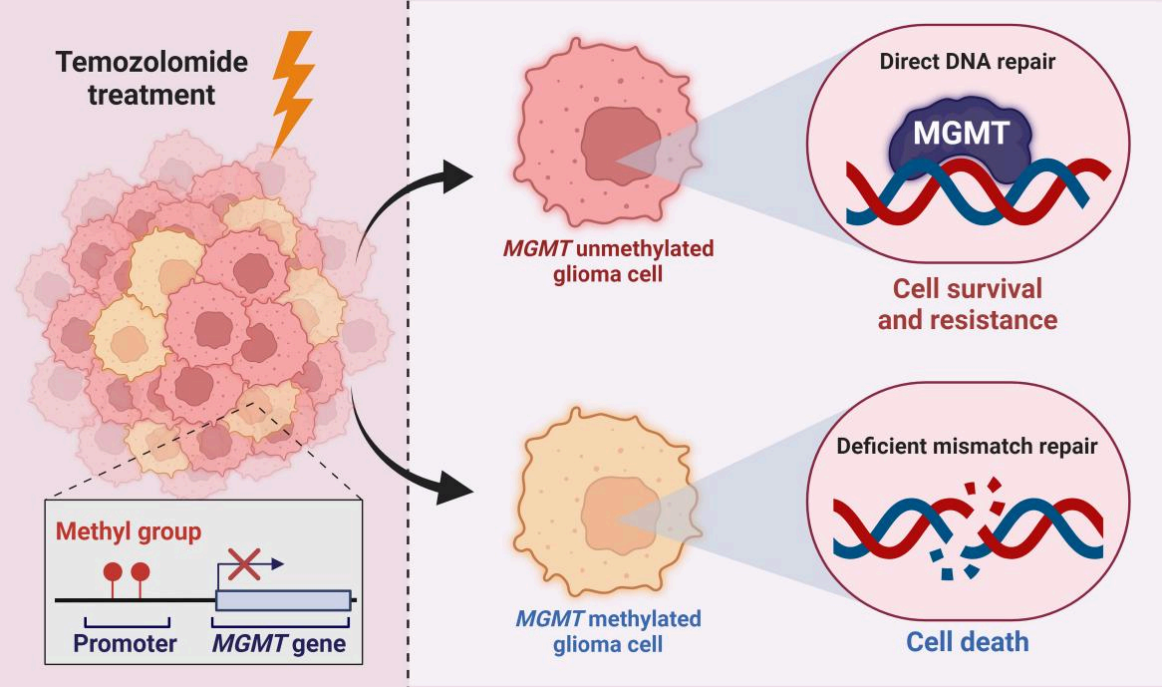
**MGMT methylation in glioblastoma**. Temozolomide treatment induces DNA damage, which can be neutralized by the expression of MGMT in glioblastoma cells, thereby restoring DNA integrity and allowing glioblastomas to evade chemotherapeutic toxicity. However, deficient mismatch repair, through MGMT methylation (i.e. deactivation), can lead to DNA strand breaks and glioma cell death.

DNA methylation is aberrant in glioblastoma. The promoter methylation status of O^6^-methylguanine–DNA methyltransferase (*MGMT*), DNA-repair gene, remains the strongest predictor of outcome and response to alkylating agents in glioblastoma.^85-87^  *MGMT* promoter hypermethylation is associated with better overall survival and better response to temozolomide therapy.^42^ Conversely, *MGMT* promoter unmethylated glioblastomas do not benefit from temozolomide and have poorer survival outcomes^42,88^ (Fig. 4).

It has been suggested that some patients with *MGMT* unmethylated glioblastoma may benefit from temozolomide, as observed in various cohorts and large-scale studies^42,89,90^; however, this is largely due to testing bias. Of note, the *MGMT* promoter consists of 97 CpG dinucleotide sites divided into three regions: R1, R2 and R3. R1 is the farthest from the transcription start site, while R3 is adjacent to it. The quantitative methylation-specific PCR (qMSP) test is most commonly used to evaluate *MGMT* methylation, focusing on the R3 region, which shows the strongest correlation with patient outcomes.^91^ However, the success of the test relies on at least four out of the seven CpG sites in R3 being co-methylated. The methylation-specific multiplex ligation-dependent probe amplification (MS-MLPA) test, on the other hand, can assess the methylation of individual CpG sites in each of the three regions (R1, R2 and R3).^92^ Therefore, both tests complement each other. When performed together, the sensitivity of detection increases, and the survival advantage seen in the unmethylated group diminishes.^93^ These findings question the assumed benefit of temozolomide for all patients with glioblastoma and underscore the need for better diagnostics and treatment regimens since the current standard of care does not provide an advantage for *MGMT*-unmethylated patients.

Nanopore sequencing, a method that detects changes in electrical current as DNA molecules pass through a nanopore, can be utilized for better characterization of *MGMT* methylation. This method enables real-time, single-molecule sequencing without the need for amplification, potentially offering advantages in longer DNA fragment sequencing and rapid data generation.^94-96^ A proof-of-concept study on 45 patients with glioblastoma showed that nanopore sequencing can accurately categorize *MGMT* methylation profiles, yielding precision akin to the EPIC array method and resolution markedly better than next-generation sequencing panel sequencing.^97^

Hypermethylation of other CpG island promoters have been identified in glioblastoma. *RB*, *CDKN2A*, *PTEN* and *TP53* have all been reportedly affected in glioblastoma. Hypermethylation of the *RB* promoter inactivates the signalling pathway and affects the response of tumour cells to CDK4/6 inhibitors.^35,36^ The expression of *p16/CDKN2* produces p16 protein that inhibits CDK4/6 and halts the cell cycle. About 50% of gliomas do not express p16/CDKN2 protein or mRNA.^98^ When the wild-type *p16/CDKN2* gene is present, it is transcriptionally repressed by aberrant hypermethylation of the CpG island. This inhibits the expression of p16/CDKN2 and leads to glioblastoma progression.^98^ The loss of PTEN expression through promoter hypermethylation activates the PI3K/AKT signalling pathway and increases the expression of the mucin-like transmembrane glycoprotein podoplanin (PDPN).^32,33^  *PDPN* promoter hypermethylation silences its expression and reduces the proliferation and migration of glioblastoma cells.^33^ The 5′ region of the *TP53* gene does not contain a CpG island; however, a basal promoter region of 85 bp is essential for its full promoter activity. Hypermethylation of the *TP53* promoter or other genes involved in the TP53 pathway, such as p14^ARF^, leads to the disruption of the TP53/p14^ARF^ pathway in several glioblastoma cell lines.^35,99^

Hypomethylation of specific DNA promoters has been associated with prognostic factors in glioblastoma. Hypomethylation and overexpression of neuromedin B was associated with improved survival outcomes.^100^ Hypomethylation and overexpression of chitinase-3-like protein 1 (*CHI3L1*), S100 calcium-binding protein A4 (*S100A4*), lysyl oxidase (*LOX*), S100 calcium-binding protein A11 (*S100A11*) were associated with poor survival outcomes.^100^ Demethylation in C-X-C chemokine receptor type 4 (*CXCR4*), T-box transcription factor 18 (*TBX18*), Sp5 transcription factor (*SP5*) and transmembrane protein 22 (*TMEM22*) were associated with tumour initiation and progression in glioblastoma.^101^

## Intratumoural distinctions inform intertumoural heterogeneity

One definition of tumoural heterogeneity is the variations observed between tumours of different cell types.^102^ In glioblastoma, cellular signatures and composition of the tumour microenvironment can correlate with prognosis and tumoural progression. Namely, the abundance of stem cells and the phenotype of immune cells can dictate outcomes. The notion that glioblastoma is not a homogeneous collection of cells has been long-established. Due to the presence of different types of cells and factors within tumours, recent scientific research has been focused on understanding the intertumoural diversity in the tumour microenvironment, i.e. intratumoural heterogeneity.

Recent studies have identified two responder subtypes in recurrent glioblastoma based on transcriptional reprogramming driven by polycomb-based chromatin remodelling. Up responders (increased gene expression post-treatment) are characterized by enrichment in proneural glioblastoma stem cells and differentiated neoplastic cells, whereas down responders (decreased gene expression post-treatment) undergo mesenchymal transition, suggesting the potential for subtype-specific therapeutic strategies.^103^ The transition towards a mesenchymal phenotype is accompanied by increased interactions with myeloid cells^104^ and increased T-cell abundance associated with hypermutation status, a process regulated by activator protein 1.^105^

### Stemness

Cellular heterogeneity in glioblastoma is dictated by the size of the glioma stem cell (GSC) populations in the tumour microenvironment. Glioblastoma tumours with higher proportions of GSCs have been associated with poorer survival outcomes.^106,107^ GSCs display high levels of cellular plasticity as they can self-renew, proliferate continuously and initiate tumour formation.^108^ They further possess intrinsic abilities to invade brain tissue, escape the immune attacks, adapt to microenvironmental stressors and initiate angiogenesis.^102,109^ These stem cell qualities allow glioblastoma tumours to resist therapy, progress and recur.^108,110,111^ Mechanisms of GSC resistance include enhanced DNA repair, diminished apoptotic signalling, anomalous DNA checkpoints and the expression of ATP-binding cassette transporters and multidrug resistance channels.^108^

**Figure 5:**
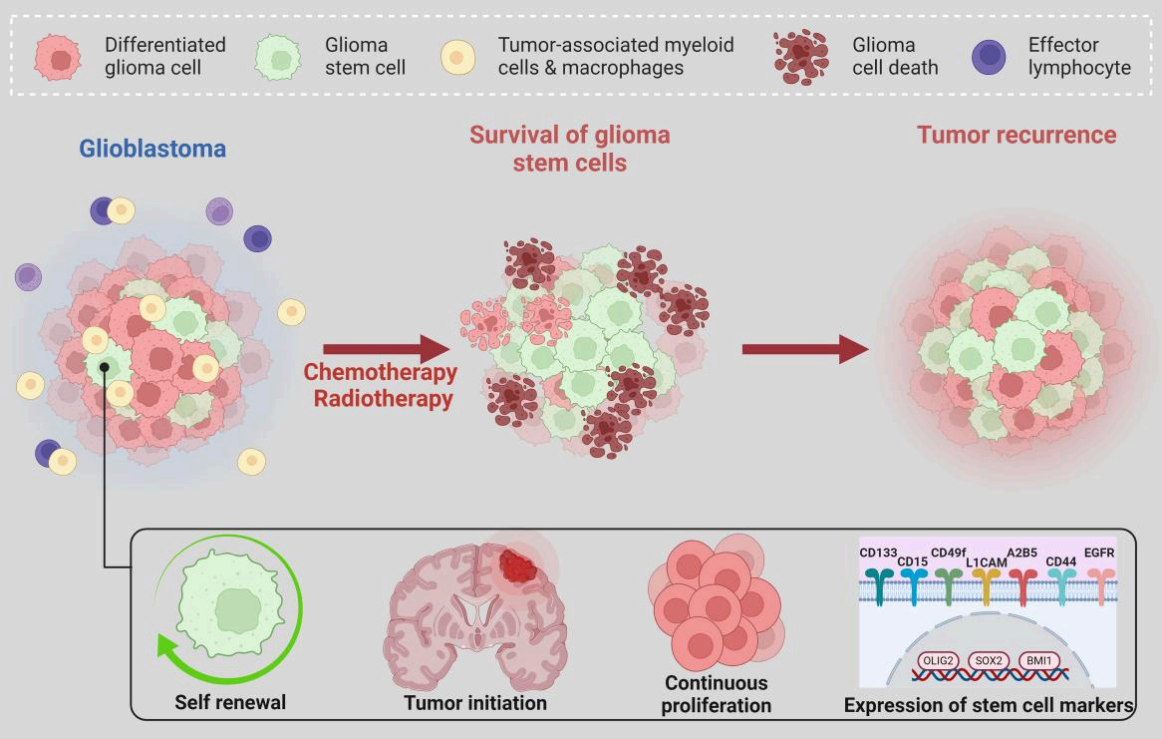
**Intertumoural heterogeneity as a function of cellular signatures in glioblastoma**. The abundance of stem cells and tumour-associated myeloid cells and macrophages predict more aggressive tumour subtypes and therapeutic resistance. GSCs are characterized by their self-renewal, tumour initiation capacity, continuous proliferation and the expression of a set of stem cell markers. Chemotherapeutic treatment of malignant gliomas can effectively target differentiated and nontumourigenic cancer cells that are highly proliferative; however, GSCs remain unaffected, which later leads to tumour relapse and recurrence.

GSCs can be distinguished from other tumour cells through established cell surface and intracellular markers. The glycoprotein prominin-1 (CD133) is used widely to identify GSC subpopulations in glioblastoma.^112,113^ CD133, a cell surface marker, contribute to the AKT and WNT signalling pathways to promote the stem cell state in the glioma cell.^108,114^ Sialyl LewisX (CD15), integrin α6 (CD49f), L1 cell adhesion molecule (L1CAM) and CD44 molecule (CD44) are other cell surface markers identified to drive GSC proliferation in glioblastoma.^108^ Sex-determining region Y-box 2 (SOX2), myelocytomatosis oncogene (MYC) and nestin (NESTIN) are intracellular protein markers that have been shown to also favour GSCs in the tumour microenvironment^108,115^ (Fig. 5).

Intercellular heterogeneity in glioblastoma can further be demonstrated through cellular growth rates. Glioblastomas grow exponentially when predominated by GSC subpopulations.^116^ Growth rate in glioblastoma is determined largely by the active proliferation of GSCs. For continuous exponential growth and expansion, GSCs migrate and accumulate at the edges of the tumour rather than in its core.^116,117^

### Immune microenvironment

Glioblastoma subtypes have been shown to have diverse immune populations. Exploring the immune cellular composition of these tumours allowed for better characterization of intertumoural heterogeneity.^118^ TAMs and myeloid cells, neutrophils and CD4+ T cells were enriched in mesenchymal glioblastoma subtypes.^41^ Activated dendritic cells were more prevalent in the classical glioblastoma subtype.^41^

TAMs comprise 30% of cells in glioblastoma.^119^ Their abundance favours tumour progression.^120,121^ While TAMs with a type 1 macrophage phenotype are traditionally pro-inflammatory and can eradicate organisms, TAMs expressing the M2 phenotype are immunosuppressive in nature.^122^ Predominance of the M2 phenotype in the tumour microenvironment has been associated with worse prognosis in IDH-wt glioblastoma.^123-126^

**Figure 6:**
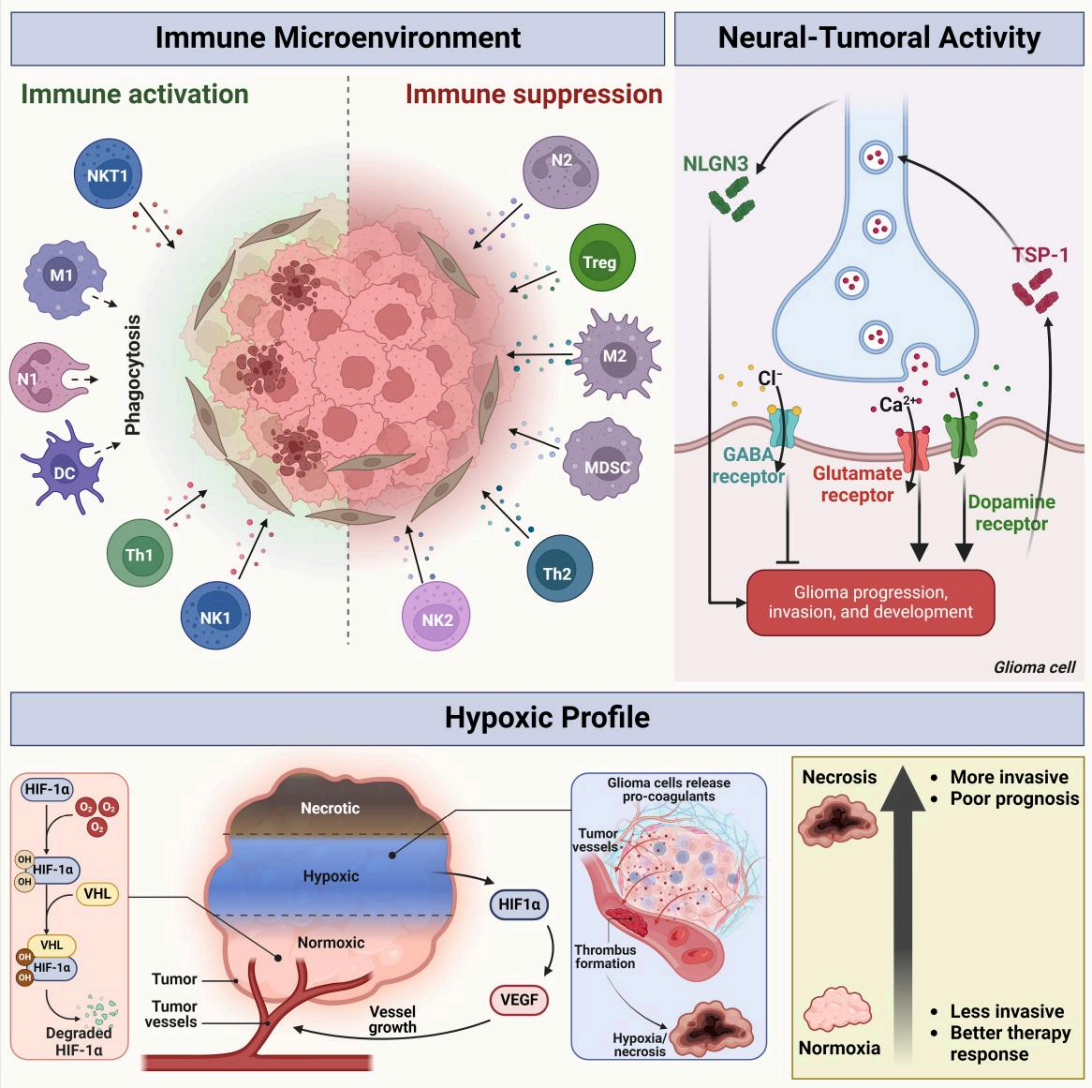
**Intratumoural heterogeneity informs intertumoural heterogeneity**. (*Upper left*) The presence of cytotoxic and effector immune cells leads to better immune responses against glioblastoma. In contrast, the abundance of tumour-associated immune cells leads to immunosuppression and increased gliomagenesis. (*Upper right*) Release of NLGN3 by neurons and glutamate and dopamine in the neuron-tumour synapses leads to calcium influx into the glioma cell, activating signalling pathways that promote glioma progression and invasiveness. Glioma cells can release TSP-1 that enhances neuronal–tumoural interactions and enforces pro-tumoural signalling. Influx of Cl^−^ through GABAergic channels into the glioma cell inhibits progression. (*Bottom*) In well perfused tumour parts, oxygenation promotes HIF1A degradation. As the glioma proliferates, it outgrows its vascularization and releases pro-coagulation factors that lead to microvascular thrombosis. This causes hypoxia and the release of HIF1A, which triggers angiogenesis and increases tumoural perfusion. Tumour cells in perinecrotic areas invade areas with better perfusion, increasing invasiveness of glioma and decreasing survival. *NKT1 (natural killer T cell type 1), M1 (type 1 macrophage), N1 (neutrophil type 1), DC (dendritic cell), Th1 (type 1 T helper cell), NK1 (natural killer cell type 1), N2 (neutrophil type 2), Treg (regulatory T cell), M2 (type 2 macrophage), MDSC (myeloid-derived suppressor cell), Th2 (type 2 T helper cell), NK2 (natural killer cell type 2), VHL (Von Hippel-Lindau) and VEGF (vascular endothelial growth factor)*.

Generally, glioblastomas exhibit a limited presence of tumour-infiltrating lymphocytes.^127^ The quantity of lymphocytes present within the tumour microenvironment corresponds to the effectiveness of immunotherapeutic treatments.^128^ A reduced amount of effector immune cells within the tumour results in a weaker response to immune stimulatory therapies, such as checkpoint blockade and vaccination.^129,130^ In addition, tumour-infiltrating cells present in the tumour microenvironment exhibit an exhausted phenotype with lower levels of activating receptors, rendering T and natural killer cell therapies ineffective^131,132^ (Fig. 6).

### Neural–tumoural interactions

Neuron–glioma synapses play a significant role in communication between glioma and neurons.^133^ These synaptic interactions can increase or decrease glioma invasiveness and are regulated by neurotransmitters, ion channels, tumour microtubes and gap junctions, which influence glioma growth.^134^ The release of gamma-aminobutyric acid (GABA), an inhibitory neurotransmitter, leads to chloride influx in the glioma cell, halting its progression and development.^135^ In contrast, a high concentration of glutamate in the tumour microenvironment can counteract GABA’s inhibitory role. Glutamate is the predominant excitatory neurotransmitter and increases the rate of Ca^2+^ influx by upregulating the Ca^2+^ permeable-α-amino-3-hydroxy-5-methyl-4-isoxazole propionic acid receptor (AMPAR) in glioma.^136^ AMPAR-mediated neuronal activity promotes tumour invasion and growth. Glutamate secretion and the downregulation of glutamate re-uptake contribute to neuronal hyperexcitability, which is implicated in glioma-associated epilepsy.^137^ Excess glutamine accumulates in the peritumoural fluid, leading to glutamate excitotoxicity and neuronal necrosis through a massive elevation of intracellular Ca^2+^ and reduction in cellular ATP levels.^138^ Neuronal death provides adjacent glioma cells more room for growth and invasion. The effect of glutamine can be countered with serotonin.^139^ Dopamine, however, increases glioma cell proliferation. Dopamine receptor D4 (DRD4) on glioma cells activates the downstream effectors PDGFRB, extracellular signal-regulated kinase 1/2 (ERK1/2) and mammalian target of rapamycin (mTOR), thereby increasing glioma stemness and tumorigenesis.^140^ DRD4 inhibition disrupted the autophagy-lysosomal pathway, leading to the accumulation of autophagic vacuoles followed by G0/G1 arrest and apoptosis.^140^

Neuroligin-3 (NLGN3) is a protein that plays a crucial role in synaptic function and maturation by binding to presynaptic neurexin. NLGN3 secretion is induced by spontaneous neuronal activity and potentiated neuronal activity leads to an increase in NLGN3 cleavage mediated by matrix metalloproteinases (MMPs).^141^ NLGN3 is positively correlated with oscillatory brain activity and negatively associated with progression-free survival of patients with glioma.^142,143^ NLGN3 activates several oncogenic signalling pathways, induces transcriptional changes and induces the expression of Tweety homologue-1, which has a role in the construction of the glioma microtube network in high-grade glioma.^144^ PKC-induced NLGN3 cleavage is dependent on MMPs, particularly, MMP3 and MMP9.^141^ Patients with glioblastoma were indicated to harbour high levels of NLGN3 in the deep regions of the brain, which may partly explain the high recurrence rate of glioblastoma. A disintegrin and metalloproteinase domain-containing protein 10 (ADAM10) inhibitors have been reported to prevent the release of NLGN3^145^ and are currently being tested in a Phase 1 clinical trial for high grade gliomas (NCT04295759).

The secretion of thrombospondin-1 (TSP-1), a synaptogenic factor, by tumour cells in functionally connected regions leads to elevated neuron–glioma interactions.^146^ Gabapentin, a U.S. Food and Drug Administration (FDA)-approved drug, effectively inhibits TSP-1 and results in reduced glioblastoma proliferation.^146^ Enhanced functional connectivity between glioblastoma and the normal brain corresponds to a decrease in patient survival and cognitive performance^146^ (Fig. 6).

### Hypoxic profile

Necrosis in the tumour microenvironment has been demonstrated to be a powerful predictor of poor patient prognosis.^147^ Many have proposed that tumours grow in a manner that attenuates local blood flow, leading to perfusion-limited hypoxia and necrosis. Evidence emerged to suggest that microscopic intravascular thrombosis within a tumour, likely caused by overproduction of pro-coagulants, triggers hypoxia and necrosis, leading to tumour microenvironment restructuring that accelerates growth^148-150^ (Fig. 6). Microscopic thrombosis is present in almost all glioblastomas but rarely found in lower grade gliomas without necrosis.^151^

HIFs are primary sensors for cellular oxygen levels, activated in response to hypoxia. HIFs promote cell survival through gene expression changes that adapt to hypoxic conditions. In malignant gliomas, HIF activation can promote disease progression through increased expression of hypoxia-induced genes, leading to a more aggressive and invasive form of glioblastoma and increased angiogenesis.^151^

Necrosis is critical in reshaping the brain tumour microenvironment. Distinguishing its effects from hypoxia can be challenging due to their intertwined relationship with glioblastoma histopathology, including intratumoural thrombosis and microvascular proliferation.^152-154^ Vaso-occlusion resulting from intravascular thrombosis leads to sustained hypoxia/anoxia, causing cellular necrosis and forcing glioblastoma cells to migrate towards a more hospitable environment.^150,155^ The hypoxic perinecrotic niche is enriched with a stem-like phenotype of glioblastoma that is more invasive and associated with poor survival^151^ (Fig. 6).

## Discussion

**Figure 7:**
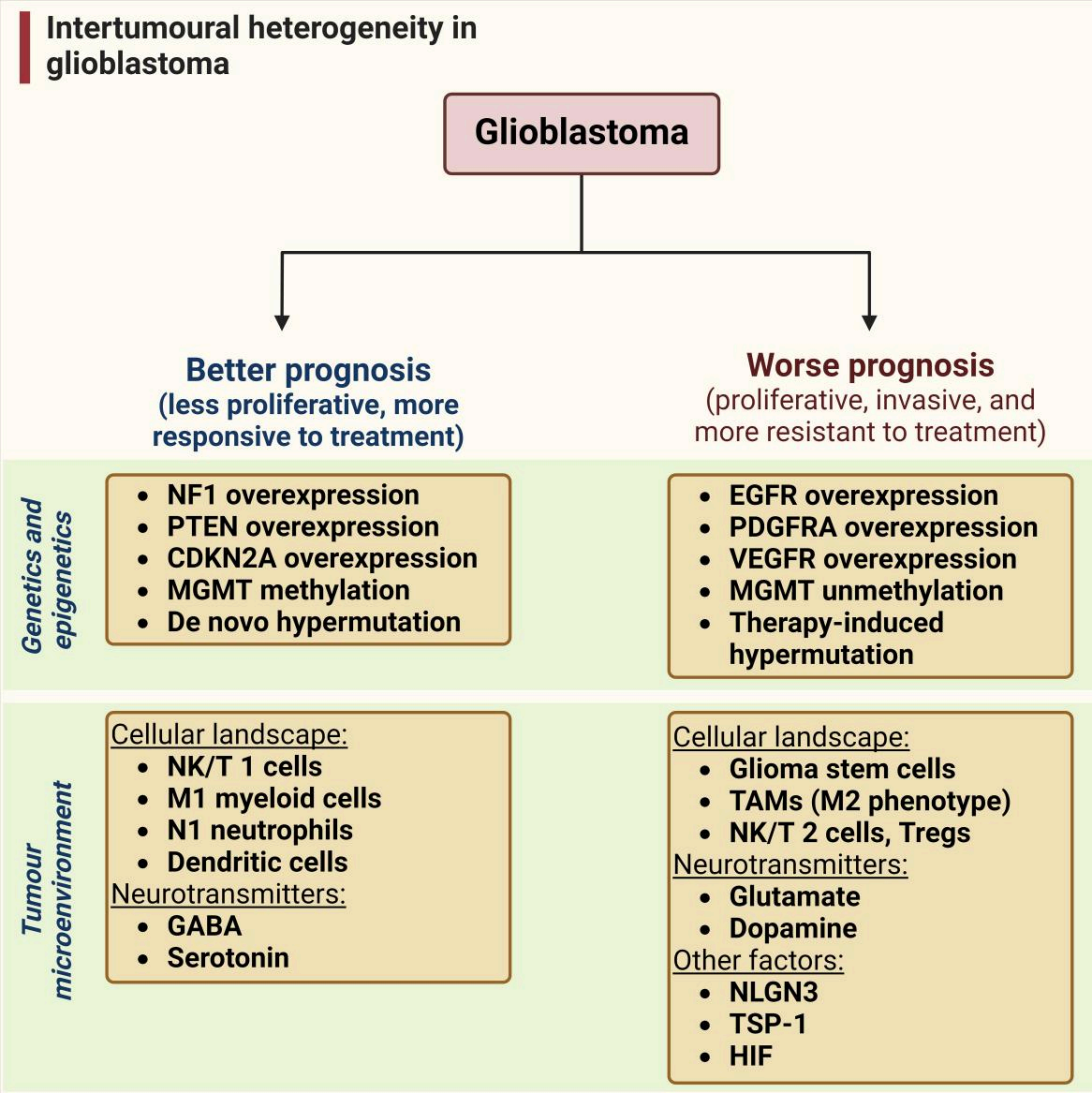
**Intertumoural heterogeneity in glioblastoma**. Preclinical and clinical investigations have shown that intertumoural heterogeneity in glioblastoma affects tumoural behaviour, response to therapy and prognostic outcomes. Overexpression of NF1, PTEN and/or CDKN2A has been associated with better prognosis in glioblastoma. Conversely, overexpression of EGFR, PDGFRA and/or Vascular endothelial growth factor receptor increases glioblastoma proliferation and leads to worse prognosis. MGMT methylation dictates response to temozolomide. While de novo hypermutation is associated with better responses to immunotherapy, therapy-induced hypermutation has been associated with worse outcomes. In the tumour microenvironment, the abundance of natural killer/T cell type 1 effector cells, type 1 macrophage-phenotype myeloid cells, neutrophil type 1-phenotype neutrophils and dendritic cells is associated with better response against glioblastoma. Conversely, the predominance of glioma stem cells, TAMs, regulatory T cells and exhausted effector cells is associated with glioblastoma proliferation and resistance to therapy. Microenvironmental factors such as GABA and serotonin have been associated with inhibitory effects on tumour growth. On the other hand, glutamate, dopamine, along with NLGN3, TSP-1 and HIF factors promote tumoural proliferation and progression.

Although updates in the 2021 World Health Organization classification have led to a more biologically homogeneous group,^156^ IDH-wt glioblastoma remains clinically and molecularly diverse (Fig. 7). Despite promising preclinical results, translating targeted therapies to the clinic remains challenging, and clinical trials may fail to identify patients who would benefit due to intertumoural heterogeneity. Recent efforts have been focused on identifying new predictive biomarkers to enhance specific treatment regimens.

Targeted therapies have shown limited success in glioblastoma, with genetic and phenotypic diversity contributing to therapeutic resistance. Enzastaurin, a selective PI3K inhibitor, has demonstrated favourable outcomes in preclinical studies.^157,158^ Nevertheless, in general, monotherapies, targeting a single gene/protein, have often failed in glioblastoma and/or led to tumour recurrence.^9^ Bevacizumab, a monoclonal antibody targeting VEGF, has largely failed to improve survival outcomes in two clinical trials in patients with newly diagnosed glioblastoma.^159,160^ Some studies had suggested that the proneural subtype may exhibit a more favourable response to bevacizumab, with potential improvements in progression-free survival and overall survival compared to the classical and mesenchymal subtypes.^161^ However, glioma cells could resist anti-VEGF inhibitors by virtue of their genetic architecture or by upregulating alternative pro-angiogenic pathways, recruiting pro-angiogenic cells to protect glioma vasculature and/or increasing invasion into neighbouring or distant cells for vascular co-option.^162^ Despite advances and continuous efforts in clinical trials,^163,164^ there has not been an agent in recent time that has shown efficacy in a double-blinded randomized controlled higher phase trial.

Immunotherapy trials on glioblastoma patients have shown limited success,^165-167^ attributed to the heterogeneity of immunosuppressive mechanisms. However, patient analysis suggests potential responders with specific mutations. Patient analysis revealed that in patients with recurrent glioblastoma, BRAF/PTPN11 mutations were enriched in 30% of those who responded to PD-1 blockade.^168^ ERK1/2 phosphorylation further predicted survival outcomes in those who received PD-1 blockade.^169,170^

IDH-wt glioblastoma with high mutational burden has been studied to understand its predictive role in immunotherapy response. High mutational burden in glioblastoma can be achieved either through a de novo pathway linked to genetic defects or a post-treatment pathway associated with acquired resistance in chemotherapy-treated glioblastoma.^171^ De novo replication repair deficient glioblastoma was found to have improved survival when treated with immune checkpoint blockade.^172^ However, post-treatment hypermutated glioblastomas have been reported to exhibit resistance to PD-1 blockade.^171^ Other studies have further shown that glioblastoma with biallelic mismatch repair deficiency display significantly high mutational and neoantigen loads and respond better to immune checkpoint inhibitors.^173^ In addition, glioblastoma with somatic POLE mutations exhibit better progression-free survival.^174^

Decoding both intertumoural and intratumoural heterogeneity is crucial for developing effective therapies. Identifying biomarkers critical for patient allocation to target trials is essential for improving glioblastoma management. Personalized strategies targeting intertumoural heterogeneity are under investigation in preclinical settings. Screening a panel of drugs on patient-derived tumour spheres^175^ or organoids^176^ helps discover therapeutics targeting unique features of each patient’s glioblastoma. Nevertheless, optimal patient recruitment to account for intertumoural heterogeneity will not be enough to achieve successful outcomes. Intratumoural heterogeneity, distinct genetic mutations and molecular pathways within the same tumour makes it difficult to target all glioblastoma cells with a single therapy. Besides, tumours may evolve over time and acquire new mutations that confer resistance to treatment. Therefore, it is essential to develop therapeutic strategies that target multiple resistance pathways and anticipate tumour evolution. Combination therapies that are designed to synergistically target meaningful biological pathways and multiple resistant pathways hold promise. Targeting universal metabolic and physiological pathways that are critical to the survival of glioblastoma cells can be another favourable approach. Furthermore, obtaining multiple biopsies during surgery, encompassing both enhancing and infiltrative nonenhancing regions allows subsequent genome-wide profiling. This informs the selection of drugs targeting actionable targets in the diffuse areas of the lesion.^177^ As such, customized interventions, tailored to the unique genetic profile of individual tumours, are essential for enhancing treatment efficacy.

The decision-making process in patient management is a critical element of glioblastoma heterogeneity. The presence of distinct subtypes, some associated with exceptionally poor outcomes, underscores the significance of shared decision-making when determining the most appropriate course of action. By incorporating comprehensive molecular profiling and subtype classification into clinical practice, healthcare providers gain a better understanding of prognostic implications, enabling informed discussions with patients about treatment options and potential outcomes. Shared decision-making empowers patients to actively participate in their care, considering risks, benefits and individual preferences, ultimately improving patient outcomes and quality of life.

## Conclusion

Glioblastomas are notoriously difficult to treat, and targeted therapies have shown limited success in changing survival outcomes. Genetic and phenotypic diversity observed between glioblastomas play a major role in driving therapeutic resistance. Despite promising results in preclinical models, translating targeted therapies to the clinic has been challenging. Clinical trials may have failed to identify patients who are most likely to benefit from the therapy due to intertumoural heterogeneity in the patient pool. Therefore, identifying biomarkers that can help allocate patients to target trials is critical to change the current status quo in glioblastoma management. Understanding and addressing glioblastoma heterogeneity at both intertumoural and intratumoural levels are essential for advancing therapeutic approaches and improving patient outcomes. Shared decision-making, integrating molecular profiling, facilitates informed choices for both healthcare providers and patients, considering risks, benefits and individual preferences.

## Data Availability

Data sharing is not applicable to this article as no new data were created or analysed.
